# Prevention of white spot lesions around orthodontic brackets using organoselenium-containing antimicrobial enamel surface sealant

**DOI:** 10.1016/j.heliyon.2021.e06490

**Published:** 2021-03-12

**Authors:** Bennett T. Amaechi, Brandon McGarrell, Minh N. Luong, Linda O. Okoye, Peter T. Gakunga

**Affiliations:** a Department of Comprehensive Dentistry, University of Texas Health San Antonio, School of Dentistry, 7703 Floyd Curl Drive, San Antonio, TX 78229, USA; b Department of Orthodontics, University of Texas Health San Antonio, School of Dentistry, 7703 Floyd Curl Drive, San Antonio, TX 78229, USA; c Faculty of Dentistry, College of Medicine, University of Nigeria, Nigeria

**Keywords:** Denteshield™, Enamel surface sealant, Orthodontics bracket, OrganoSelenium, White spot lesions

## Abstract

**Objectives:**

To investigate the antimicrobial potential of organo-selenium compound when applied as enamel surface sealant or primer (DenteShield™ [DS]) around orthodontic brackets to prevent enamel demineralization.

**Methods:**

Human teeth were randomly assigned to seven treatment groups (15/group): control (No primer or sealant), Leopard light primer (LLP), DS Primer (DS-P), DS Enamel Surface Sealant (DS-S), Pro Seal, Opal Seal and combined DS-P/DS-S (DS-PS). Following etching, the tooth surface was coated with their respective material (except control group) and a bracket was bonded on each treated surface. All samples were subject to cariogenic challenge in a continuous flow microbial caries model at 37 °C in an incubator for 28 days. Demineralization was evaluated with Transerse microradiography to determine mineral loss (Δz) and lesion depth (LD). Data was statistically analyzed using Bonferroni protected Mann-Whitney tests (α = 0.05).

**Results:**

Demineralization was obsessrved only in Control and LLP groups. Control group had significantly (p < 0.001) greater mean LD (109.47 ± 34.22 μm) and mean Δz (2251.07 ± 514.26 vol%μm) when compared with the LLP with mean LD (44.98 ± 11.69 μm) and Δz (700.67 ± 310.66 vol%μm). All other groups did not develop any lesions.

**Conclusions:**

Selenium-based primer and sealant used alone or in combination were effective in protecting enamel from demineralization around brackets. The combination of primer and enamel surface sealant has no added benefit.

**Significance:**

DS-S and DS-P containing antimicrobial organo-selenium compound can prevent whitespot lesions development when applied on tooth surface during orthodontic treatment. Light primer applied alone on tooth surface may not provide adequate protection for the enamel around orthodontic appliances.

## 1 Introduction

With the increasing demand for esthetics in dentistry, the treatment options have been drastically diversified including veneers, biomimetic dentistry, tooth bleaching, implants and periondontal surgery, orthognathic surgery, and orthodontics. Orthodontics, combined with other treatment modalities, could result in change to the patient's teeth alignment and occlusion, but also the soft and hard tissue reaching a dentofacial harmony. However, a drawback during the course of orthodontic treatments is the development of white spot lesions (WSL) [1,2]. The incidence of WSL was reported to be higher in orthodontic patients than the general public [3]. The presence of bracket creates a platform that accumulates plaque on the surface of a tooth. The physical barrier from the combination of bracket, the wire and ligature hinders the self-cleaning mechanism of musculature and saliva as well as the maintenance of oral hygiene [1,4]. Studies have shown that orthodontic treatment increases the plaque formation with the significantly lower pH compared to those of non-orthodontic patients [5]. The low pH of plaque inhibits enamel remineralization and promotion demineralization, which leads to WSL.

Once the WSL develop during the treatment, the prognosis is guarded as the integrity of the enamel surface is compromised and it might require further restorative procedure after the removal of the brackets. Therefore, preventing WSL is important for a comprehensive and successful outcome of orthodontic treatment. The conventional methods of prevention for such lesion are oral hygiene procedures, diet modification, and frequent application of fluoride and antimicrobials [6,7,8]. However, the effectiveness of these methods varies [6,7,8]. Althought patient education is foremost, extensive length of orthodontic treatment time does not guarranty the compliance of patient with preventive measures throughout the period [7]. Therefore, an ideal preventive measure would require minimal patient's compliance, uncompromised esthetic and no additional chairside time [9].

The application of fluoride-releasing light-cured resin sealant around and beneath the brackets has exhibited less wear and more effectiveness in demineralization inhibition without compromising the bond strength of orthodontic brackets [10,11]. Nonetheless, a sharp decrease in fluoride ion released from the resin over the first few weeks after application have largely affected the capacity of inhibiting WSL [12,13]. Recently, the incorporation of organo-selenium into resin sealant for WSL prevention has proved to have immense potential. Organo-selenium containing pit and fissure sealant has been reported to effectively prevent plaque accumulation over and around the sealant and to prevent demineralization around the sealant [14]. Organo-selenium is found in neutrophils and NADPH oxidase and produces non-toxic radicals that inhibit cellular growth [14,15]. The Organo-selenium can be used in multi-modes in orthodontic armamentarium, such as coating on brackets, ligature ties, power chain or incorporated into adhesives, sealants and cements. Organo-selenium containing enamel surface resin sealant provides two-fold protections, physical barrier to acid attack and antibacterial action to inhibit adhesion of microorganism onto tooth surface to form plaque.

In orthodontics, antibacterial sealant DenteShield™ (DS; SelenBio Inc, Austin, TX, USA) has been used to prevent WSLs during orthodontic treatment. It served as primer and anti-demineralizeation sealant without affecting the shear bond strength of the bracket [16]. However, the method of application and its effectiveness have not been fully determined. The primary objective of the present study was to investigate the potential of using an effective concentration of the organo-selenium compound with antimicrobial action applied as resin sealant or primer (DenteShield™ enamel surface sealant and primer) around orthodontic appliances to prevent WSL formation. Specifically, we compared the anticaries efficacy of DenteShield™ sealant and primer to those of other commercial available products, using different application modes and materials around orthodontic brackets. The secondary objective was to determine the most effective method of application of these materials in orthodontic practice. Our null hypothesis was that there are no differences in the amount of demineralization between the experimental groups as measured by amount mineral loss and lesion depth.

## 2 Materials and methods

### 2.1 Teeth preparation and experimental grouping

Fifty-three sound human molars and premolars extracted due to orthodontic treatment or impaction were collected under the guidelines approved by our Institutional review Board (IRB Approval: HSC2008233N). The teeth were cleaned of debris and stains before examination using fiber-optic transillumination to exclude teeth with pre-existing WSL. Each tooth was sectioned vertically in mesiodistal direction using a water-cooled diamond saw (Beuhler, Switzerland) to produce 2 sections, each bearing either the buccal or the lingual surface of the tooth. A total of 106 sections were produced from the 53 teeth, and 105 were used and allocated randomly to seven experimental groups (15/group) based on the materials used (Table 1): Group 1: Control group - No Sealant or Primer; Group 2: Leopard Light Primer (LLP); Group 3: DenteShield™ Primer (DS-P); Group 4: DenteShield™ Enamel Surface Sealant (DS-S); Group 5: Reliance Pro-Seal™ (RPS); Group 6: Ultradent Opal-Seal™ (UOS); Group 7: DenteShield™ Primer & Enamel Surface Sealant (DS-PS).

**Table 1:** Mean values of Mineral loss (Δz, vol%.μm) in each experimental group. There was a significant difference (p < 0.001) between the Control and Leopard Light Primer.

Group	N	Minimum	Maximum	Mean ± SD	Variance
Control	15	1370	2860	2251.07 ± 514.26	264466.35
LPP	15	260	1300	700.67 ± 310.66	96506.67
SD-P	15	0	0	0.00	0.00
SD-S	15	0	0	0.00	0.00
RPS	15	0	0	0.00	0.00
OUP	15	0	0	0.00	0.00
SD-PS	15	0	0	0.00	0.00

### 2.2 Bracket bonding and enamel surface treatment

The brackets were bonded to each tooth following the current clinical procedure. The entire facial surface of the tooth was etched with 37% phosphoric acid for 30 s and then rinsed and dried using the dental air-water syringe. For the control group, Transbond® XT light-cure adhesive paste (3M Unitek, St Paul, MN) was placed on the bottom of an orthodontic bracket (*lower incisor brackets*), and the bracket bonded to the center of the etched tooth surface, and cured with LED light (Ultradent Products, Inc, USA) for 20 s. For the LLP, DS-P, DS-S, RPS and UOS, following etching and drying, each material was painted on the entire etched surface of their respective tooth samples. Then the bracket was bonded and cured as described for the control group. For the DS-PS group, following etching and drying, DS primer was coated on the entire facial surface of each tooth, and the bracket was bonded and cured as described for the control group. Following bracket bonding, DS enamel surface sealant was coated on remaining facial surface of the tooth around the bracket, and light cured with LED light for 20 s.

### 2.3 Demineralization procedure

The experiment was conducted in an Artificial Mouth, which is a continuous flow biofilm model, housed inside a CO_2_ incubator maintained at a constant physiological temperature of 37 °C [17,18]. The Artificial Mouth was previously developed in our laboratory and validated in previous studies [17,18]. Prior to the experiment, all components of the Artificial Mouth and the specimen were sterilized using ethylene oxide gas. The artificial mouth is a multiple-chamber system where each experimental group was assigned to a chamber. The specimen were embedded in the grooves on the surface of the cylindrical rod in the chamber using heavy duty putty to create a block. The blocks were embedded in the manner such that their surfaces flushed with the surface of the cylinder to permit streamlined flow of fluids, and the exposed enamel could be susceptible to plaque growth and subsequent demineralization. Caries development on the tooth surfaces was initiated by circulation of Todd Hewith broth inoculated with multispecies inoculum of *Streptococcus mutans* (NCTC 10449) and *Lactobacilli casei* (NCIB 8820) culture (broth to inoculums ratio 10:1) through the chambers for 4 h (adhesion phase). The Todd Hewitt broth was enriched with reduced glutathione (150 μM) to provide thiols that is present in the human saliva, and which is needed for generation of superoxide radicals. Following 4 h of adhesion phase, the inoculated Todd Hewith broth was replaced with a bacteria-free fresh Todd Hewitt broth, and this was continuously circulated through the four chambers to nutritionally simulate saliva, while 5% sucrose was supplied every 6 h for 6 min to simulate the daily meal intake. All fluids, including inoculation, was delivered at a flow rate of 0.3 ml/min (average unstimulated salivary flow rate). Change in plaque pH following sucrose supply was monitored after 24 h (biofilm maturation phase) to confirm exhibition of Stephan's curve (plaque pH curve) under sucrose challenge. The experiment lasted for 28 days, and the tooth blocks were harvested and processed for demineralization assessment using Transverse Microradiography (TMR).

### 2.4 Transverse microradiography and image analysis

Following the 28 days exposure to dental plaque, TMR was used to evaluate any demineralization of the tooth specimens. The specimen were sectioned into thin slices of approximately 150 μm thick using water-cooled diamond wire saw (Buehler, Germany). Each slice was polished to reduce the thickness of the slice to 80–100 μm (the appropriate thickness for TMR) using Adhesive Back 6 μm lapping film in a MultiPrep™ Precision Polishing machine (Allied High Tech, USA). Both sides of the slice were polished to achieve planoparallel surfaces. Then the slices were microradiographed on type lA high resolution glass X-ray plates (Microchrome Technology, CA, USA) using a Phillips x-ray generator system (Panalytical, Amsterdam) set up for this purpose. The plates were exposed for 10 min at an anode voltage of 20kV and a tube current of 10 mA, and then processed. Processing consisted of a 5 min development in Kodak HR developer and 5 min fixation in Kodak Rapid-fixer before a final 30 min wash period. After drying, the microradiographs were examined with a Leica DMR optical microscope linked via a Sony model XC-75CE CCTV camera to a Computer housing the image analysis program (TMR2006 version 3.0.0.6, Inspektor Research, Amsterdam). The enhanced image of the microradiographs were analyzed under standard conditions of light intensity and magnification and processed, along with data from the image of the step wedge, by the TMR program. The computer program calculated the parameters of integrated mineral loss (Δz, vol%.μm) and the lesion depth (LD, μm) based on the work described by De Josselin de Jong et al. (1987) [19].

### 2.5 Power analysis and sample size calculation

The sample size calculations, which were based on a power analysis, were performed using nQuery Advisor software (Statistical Solutions, Cork, Ireland). Based on previous studies in which the protection of tooth surface by different demineralization inhibition sealants (including PS) were compared, the mean difference in protective outcome measure between PS and the next protective sealant was equal to 22.5 with a standard deviation equal to 15.3 [20,21]. For the hypothesis that there would not be a significant difference in the amount of demineralization between the experimental groups as measured by amount of mineral loss and lesion depth, an effective sample size of 15 samples will have power greater than 0.80 with a 0.05 one-sided significance level to detect a difference in mineral loss and lesion depth between the groups.

### 2.6 Statistical analysis

The data of integrated mineral loss and lesion depth were subjected to normality test and Levene's variance homogeneity, showing the normal distribution and homogenity of variance. Statistical analysis of the data was performed with SPSS (version 14.0, Chicago Illinois) with the level of significance (α) pre-chosen at 0.05. For the TMR data (mineral loss & lesion depth), Bonferroni protected Mann-Whitney tests was used to identify pairwise differences in the level of demineralization among the seven groups after 28 days of cariogenic challenge.

## 3 Results

**Figure 1:**
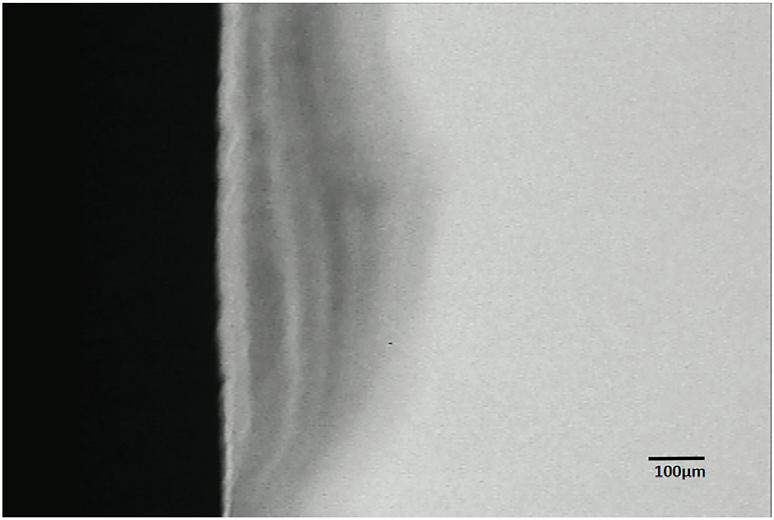
Representative microradiographic image of enamel subsurface lesions (Initial caries lesions) in samples from the control group protected with neither primer nor sealant.

**Figure 2:**
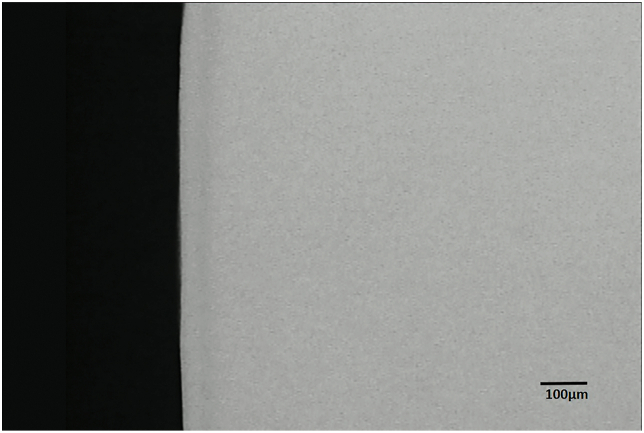
Representative microradiographic image of enamel subsurface lesions (Initial caries lesions) in samples from the group protected with Leopard Light Primer.

**Figure 3:**
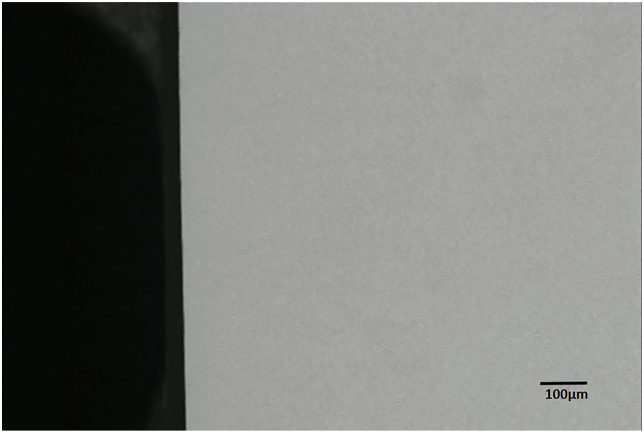
Representative microradiographic image of samples from the groups protected with DenteShield™ Primer; DenteShield™ Enamel Surface Sealant, Reliance Pro-Seal™, Ultradent Opal-Seal™, and DenteShield™ Primer & Enamel Surface Sealant.

The demineralization around the brackets was detected and measured under TMR, and the representative microradiographs from the groups can be seen in Figure 1 through 3. With sealant applied, the enamel surface was protected against demineralization by bacteria acid (Figures 2 & 3). TMR analyses from Tables 1 and 2 show that only the Control and Leopard Light Primer (LLP) groups developed WSL lesions. The Control group had significantly (p < 0.001; Bonferroni test) greater mean lesion depth (109.47 ± 34.22 μm) and mean mineral loss (2251.07 ± 514.26 vol%μm) when compared with the LLP with mean lesion depth (44.98 ± 11.69 μm) and mineral loss (700.67 ± 310.66 vol%μm). All other groups did not develop any lesions and had no mineral loss (Tables 1 and 2).

**Table 2:** Lesion depth in μm of each experimental group. There was a significant difference (p < 0.001) between the Control and Leopard Light Primer.

Group	N	Minimum	Maximum	Mean	Std Deviation
Control	15	40.70	168.30	109.47	34.22
LPP	15	27.50	64.90	44.98	11.69
SD-P	15	0	0	0.00	0.00
SD-S	15	0	0	0.00	0.00
RPS	15	0	0	0.00	0.00
OUP	15	0	0	0.00	0.00
SD-PS	15	0	0	0.00	0.00

## 4 Discussion

Orthodontic treatment has been on increase due to the dramatic increase in demand for esthetics in dentistry. However, one sequel of orthodontic treatment is the development of WSL around the periphery of the orthodontic brackets, which is the early stage of dental caries due to enamel demineralization by organic acids, a byproduct of bacteria metabolism^1^. The WSL, the prevalence of which ranges from 2 to 96%, creates an esthetic problem for the patient at the conclusion of the orthodontic treatment [22,23,24] Thus the main objective of the present study was to evaluate the potential use of an effective concentration of the organo-diselenide compounds with antimicrobial action applied as sealant or primer (DenteShield™ sealant or primer) around orthodontic appliances to prevent WSL formation. This was conducted using an established continuous flow biofilm model (microbial caries model) acting as an artificial mouth [17,18]. The tooth samples were subjected to the natural demineralization in the presence of plaque biofilm frequently fed with sucrose without toothbrushing, equivalent to plaque accumulation around brackets seen during orthodontic treatment or poor oral hygiene. Thus the samples were tested under high caries risk condition. The result demonstrated that selenium-based primer and sealant offered 100% protection against tooth surface demineralization, and their protection was comparable to that of the two other commercially existing products, Opal-Seal™ and Pro-Seal™ (Tables 1 and 2 & Figure 3). However, the control group with neither primer nor sealant protection and the Light Primer group allowed significant demineralization of the tooth tissue as measured by the mineral loss and lesion depth. Thus the null hypothesis was rejected as there was a significant difference in lesion depth and mineral loss between the control group and the other groups, including the LLP. It is believed that while the four protective materials (primer and sealants) offered physical protection of the tooth against demineralization due their resin component, the selenium-based primer and enamel surface sealant have additional antimicrobial protection against plaque formation. This antibacterial effect is attributed to the organo-selenium molecule component in the DenteShield™ materials, which acts as a catalytic generator of superoide radicals from the oxidation of thiols present in the human saliva [25], but added as reduced glutathione into the growth media (Todd Hewitt broth) in the present study. The superoxide radical cause oxidative stress that damages the bacterial cell wall and DNA, thus it is toxic to different oral microorganism and kills bacteria on contact [26,27] Therefore selenium-contaning materials can prevent the development of bacterial biofilm around the brackets during orthodontic treatments, thereby preventing WSL formation [16].

The effectiveness of anticaries sealants depends on the longevity of the sealant. Although the combination of selenium-based primer and Enamel Surface Sealant may offered a thicker protective layer, the protection observed in this group is comparable to that of other sealants, so there was no special advantage with this method of application. However, it is envisaged that this combination may offer advantage in the length of protection by increased resistance to wear by toothbrush abrasion. This is being investigated in a separate study. However, based on the result of this study, the dentists therefore may apply only primer or sealant alone and not both for protection against WSL formation. It is important to mention that the selenium-based primer is composed mainly of triethylene glycol dimethacrylate (TEGDMA) and bisphenol A diglycidyl ether dimethacrylate (bis-GMA), and it is not subject to hydrolysis, however, it can be removed by toothbrushing abrasion during and after orthodontic treatment. The manufacturer advised on periodic monitoring for primer or sealant deficient areas and to have the deficient areas re-coated for adequate protection. One may be anxious about the long term color stability of the primer when using it as a protective coating. The color stability has not been tested in any study, however, it is a colorless resin, and the prime is removed by toothbrushing abrasion shortly after orthodontic treatment. However, it would be of interest to investigate the color stability in our next study.

It is pertinent to mention that selenium forms a covalent attachment to resin polymers, and as such does not leach out to the surrounding and has long-term retention of its antibacterial effects [14]. This is an advantage of the selenium-based sealant and primer over other tooth surface protective sealants such as the fluoride-releasing sealants and adhesives, which studies have shown their fluoride content to exhaust over a period of time [20,28].

It was not surprising that RPS and UOS inhibited tooth surface demineralization considering that RPS is a light-cure, fluoride-releasing, filled-resin sealant whereas UOS is glass-ionomer, fluoride-releasing nano-filled resin sealant. This observation of RPS demineralization inhibition in the present study is in agreement with previous study that reported a complete inhibition of demineralization with the use of RPS [10]. Another study reported 72% demineralization reduction when using RPS with high durability and continuous effectiveness over in vitro tooth brushing period [20]. While the present study showed a comparable efficacy with the two sealants, previous study reported that RPS released a significantly higher amount of calcium compared to UOS to provide more enamel protection. This difference was explained by the less filler content of UOS that resulted in the loss of sealant over time [29].

## 5 Conclusions

Selenium-based primer and sealant used alone or in combination were effective in preventing demineralization of tooth tissue around the orthodontics brackets. The combination of primer and enamel surface sealant has no additive effect on protection of enamel in the present study.

## Declarations

### Author contribution statement

Bennett T. Amaechi: Conceived and designed the experiments; Wrote the paper.

Brandon McGarrell: Performed the experiments.

Minh N. Luong: Contributed reagents, materials, analysis tools or data; Wrote the paper.

Linda O Okoye: Analyzed and interpreted the data.

Peter T. Gakunga: Analyzed and interpreted the data; Wrote the paper.

### Funding statement

This research did not receive any specific grant from funding agencies in the public, commercial, or not-for-profit sectors.

### Data availability statement

Data will be made available on request.

### Declaration of interests statement

The authors declare no conflict of interest.

### Additional information

No additional information is available for this paper.
